# Diel niche variation in mammalian declines in the Anthropocene

**DOI:** 10.1038/s41598-023-28104-2

**Published:** 2023-01-19

**Authors:** Daniel T. C. Cox, Alexandra S. Gardner, Kevin J. Gaston

**Affiliations:** Environment and Sustainability Institute, University of Exeter, Penryn, Cornwall, TR10 9FE UK

**Keywords:** Ecology, Biodiversity, Conservation biology, Macroecology

## Abstract

Biodiversity is being eroded worldwide. Many human pressures are most forcefully exerted or have greatest effect during a particular period of the day. Therefore when species are physically active (their diel niche) may influence their risk of population decline. We grouped 5032 terrestrial extant mammals by their dominant activity pattern (nocturnal, crepuscular, cathemeral and diurnal), and determine variation in population decline across diel niches. We find an increased risk of population decline in diurnal (52.1% of species), compared to nocturnal (40.1% of species), crepuscular (39.1% of species) and cathemeral (43.0% of species) species, associated with the larger proportion of diurnal mammals that are primates. Those species with declining populations whose activity predominantly coincides with that of humans (cathemeral, diurnal) face an increased number of anthropogenic threats than those principally active at night, with diurnal species more likely to be declining from harvesting. Across much of the land surface habitat loss is the predominant driver of population decline, however, harvesting is a greater threat to day-active species in sub-Saharan Africa and mainland tropical Asia, associated with declines in megafauna and arboreal foragers. Deepening understanding of diel variation in anthropogenic pressures and resulting population declines will help target conservation actions.

## Introduction

Defaunation in the Anthropocene is altering major macroecological patterns that have characterised life on earth^1,2^. Associated population declines have led to roughly one-fifth of the world’s extant vertebrate species being threatened with extinction^1,3,4^. However, a focus on species extinctions (the complete loss of species) has underestimated the scale of the problem because for each actual extinction serious declines in abundance in extant populations are estimated to be ~ 10 times more frequent^5^. Indeed, the Living Planet Index has indicated a decline of 67% in ~ 17,000 vertebrate populations since 1970^6^, a figure that may be an underestimation of the true extent of declines^7^.

The time of day at which an organism is physically active (its diel niche) may influence its level of exposure to many anthropogenic pressures and so its risk of population decline. First, a species' diel niche drives variation in functional traits^8–10^ that themselves are important determinants of extinction risk^11,12^. Second, individuals are often more vulnerable to threats when active than when hidden during periods of rest. Third, anthropogenic pressures may vary across the daily cycle. Humans are largely a diurnal species and as such disturbance, harvesting (e.g., hunting, poaching, snaring) and human-wildlife conflict may be more important in day-active species, while increasingly high daytime temperatures associated with global warming disproportionately impact heat and water budgets for species active during the daytime^13,14^. In contrast, the nighttime is warming faster than the daytime, allowing novel species to invade the night^15^. Given the above, the nighttime may also provide non-native species an advantage to establish and compete with native fauna^16,17^. Few pressures act in isolation, and threats such as habitat loss and harvesting^18,19^ or habitat loss and climate change^20^ may be additive or synergistic, therefore species active during periods that expose them to multiple anthropogenic threats may have an inflated risk of population decline.

Here, across 5032 extant terrestrial mammal species, we examine whether diel niche is an important, but previously overlooked, functional trait associated with population decline. We categorised species as nocturnal, crepuscular, cathemeral or diurnal before identifying which species are experiencing declining population trends and the anthropogenic threats associated with these declines. We explore four primary research questions: (1) Is there variation across diel niches in the proportion of species with declining populations? (2) Does the number of threats faced by species with declining populations vary with diel niche? (3) Is there an increased risk of population decline from the five most prevalent anthropogenic threats (habitat loss, harvesting, conflict, climate change, non-native species) dependent on when species are active? (4) Proportionally, where are diel niche communities experiencing the greatest declines from specific threats? The results of this analysis may deepen understanding of the processes underlying population declines and for developing improved assessments of species’ vulnerability, subsequently to inform conservation action.

## Results

### Diel variation in population decline

**Figure 1:**
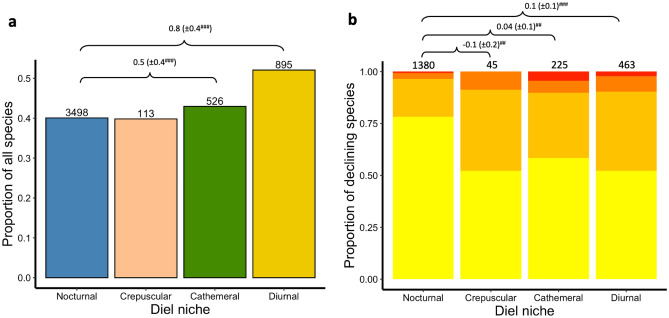
Drivers of population decline across diel niches. We show (**a**) the proportion of species in each diel niche with a declining population trend, and (**b**) the proportion of species with declining populations in each diel niche facing one (yellow), two (light orange), three (dark orange) or four (red) anthropogenic threats. We modelled the response against diel niche, where nocturnal is the base factor level and so positive parameter estimates show that a greater proportion of species are declining or face an increased number of threats in that diel niche. To account for uncertainty about phylogenetic topology or divergence dates in any one tree, we repeated each phylogenetic generalised linear model 100 times each with a different phylogenetic tree, and present the mean parameter estimates across models, the standard deviation of the parameter estimates across models is shown in parentheses and the robustness of the results is shown where ^###^ indicates a statistical significance < 0.01 in ≥ 90% of models and ^##^ in ≥ 75% and < 90% of models (Table S2). The numbers above each bar give the number of species in each diel niche for (**a**) all species, and (**b**) those with declining population trends only (Table S1).

For 5,032 extant terrestrial mammal species we obtained data on their diel niche, IUCN population trend status^21,22^ and current range distributions^23^. We found that populations were declining in 40.1% of nocturnal (*N* = 3498), 39.8% of crepuscular (*N* = 113), 43.0% of cathemeral (*N* = 526) and 52.1% of diurnal (*N* = 895) mammal species (Fig. 1a; Table S1a). Although a higher frequency of population decline is occurring in diurnal species, phylogenetic logistic regression reveals a very low *pR*^2^, and suggests that the difference is due in large part to the higher proportion of diurnal mammals that are primates than in other diel niches (32.1% of diurnal compared to 4.0% of cathemeral and 3.3% of nocturnal mammals; Table S2a). Sensitivity tests reveal no difference between diel niches in the proportion of declining species when primates and non-primates are treated separately (Appendix S1; Fig. S1).

### Number of threats faced by diel niche

Population declines in those species active during daylight, either partly (cathemeral) or fully (diurnal), were more likely to be associated with a greater number of anthropogenic threats than in those active solely at night (nocturnal) or during twilight (crepuscular; Fig. 1b; Table S1b). Where more than one threat exists, habitat loss and harvesting are the drivers most commonly associated with population decline. Diurnal primates face a greater number of threats than those active at night (Tables S1b, S2b; Fig. S2a). Across non-primates, those species with declining populations that are active during the daytime face an increased number of threats, particularly Artiodactyla (even-toed ungulates; cathemeral, diurnal), Perissodactyla (odd-toed ungulates, cathemeral) and Carnivora (cathemeral; Tables S1b, S2b; Fig. S2b).

### Population declines from main threats

**Figure 2:**
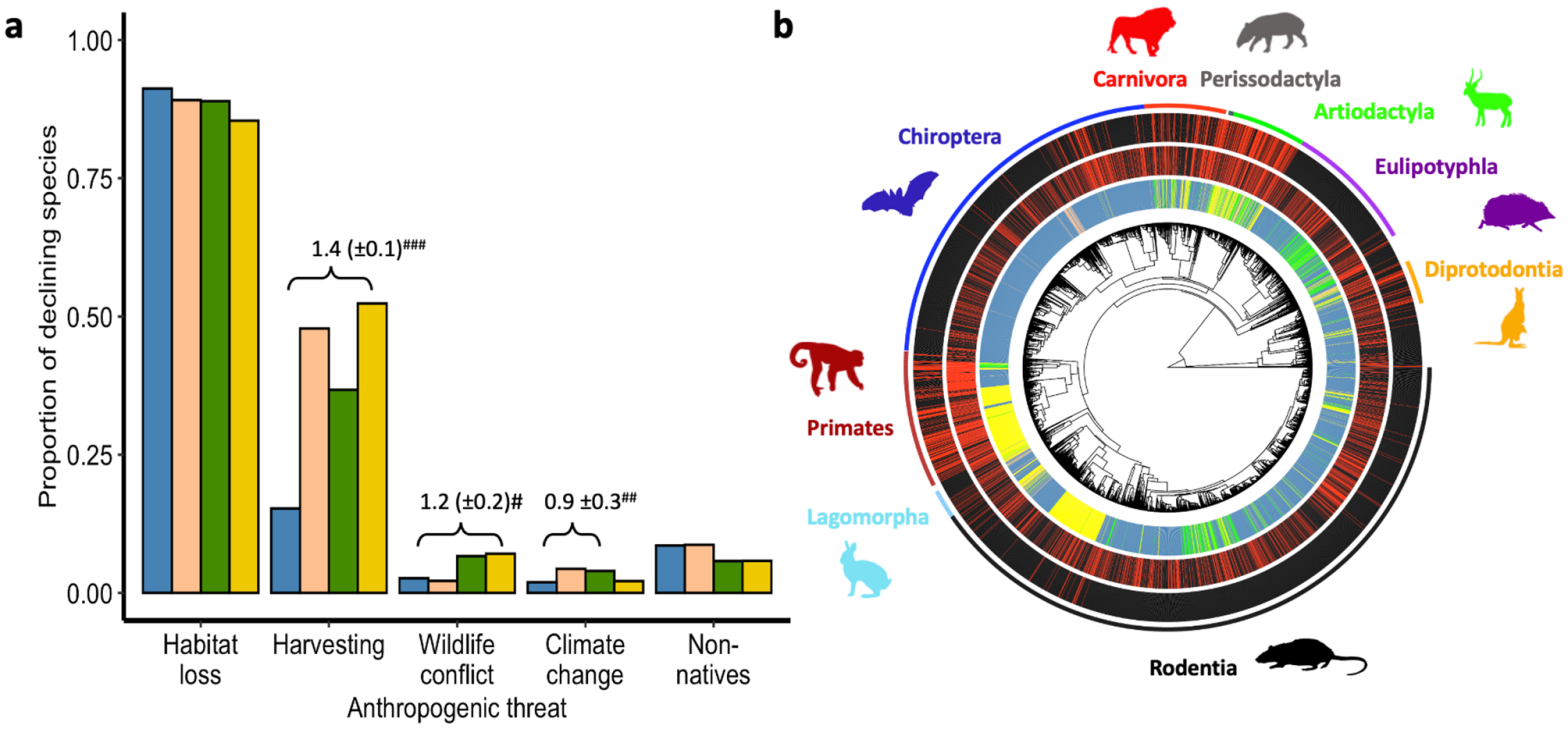
Population declines in mammals across diel niche by anthropogenic threat. (**a**) For mammals with declining populations we show the proportion that are declining due to each of the five most prevalent anthropogenic threats (nocturnal, blue, *N* = 1402; crepuscular, peach, *N* = 45; cathemeral, green, *N* = 226; diurnal, yellow, *N* = 466; Table S5). For each anthropogenic threat in turn we modelled whether a species was declining from that threat, against diel niche. Where nocturnal was the base factor level and so positive parameter estimates show that a greater proportion of species are declining in that diel niche. To account for uncertainty about phylogenetic topology or divergence dates in any one tree, we repeated each phylogenetic logistical regression 100 times each with a different phylogenetic tree, and present the significant mean parameter estimates across models, the standard deviation of the parameter estimates across models is shown in parentheses and the robustness of the results is shown, where ^###^ indicates a statistical significance < 0.01 in ≥ 90% of models, ^##^ in ≥ 75% and < 90% of models and ^#^ in ≥ 60% and < 75% of models (Table S3). (**b**) Phylogenetic tree showing 5032 species. The inner coloured radial bar indicates diel niche (nocturnal (blue), crepuscular (peach), cathemeral (green), and diurnal (yellow)), the inner middle radial bar whether the species is declining (red) or not (black) due to habitat loss, the outer middle radial bar whether the species is declining (red) or not (black) from harvesting and the outer radial bar shows selected clades. Silhouettes were freely downloaded from PhyloPic: www.phylopic.org, under CC0 1.0 Public Domain Dedication.

Harvesting is disproportionately associated with population declines in diurnal primates and non-primates (51.7% of all diurnal species with declining populations) compared to other diel niches (*pR*^2^ = 0.46; Fig. 2a; Table S1c), with diurnal and cathemeral primates and carnivores being particularly vulnerable (Figs. 2b and S3). Habitat loss was associated with declines in between 85.5% (diurnal) and 91.2% (nocturnal) of species with declining populations (Fig. 2a; Table S1c) and occurs in species from across the mammalian phylogenetic tree (Fig. 2b). Although phylogenetic logistic regression revealed that a greater proportion of nocturnal than cathemeral and diurnal species were declining due to habitat loss, the very low *pR*^2^ suggests little influence of diel niche (Fig. 2a; Table S3). Population declines associated with human-wildlife conflict, climate change and non-native species were less common (< 8% of declining species; Table S1c). However, day-active mammals were more likely to be declining from human-wildlife conflict (diurnal) or climate change (cathemeral) and less likely to be declining from non-native species (cathemeral, diurnal) than nocturnal mammals (Fig. 2a; Table S3).

### Biogeography of proportional declines in diel niche communities

**Figure 3:**
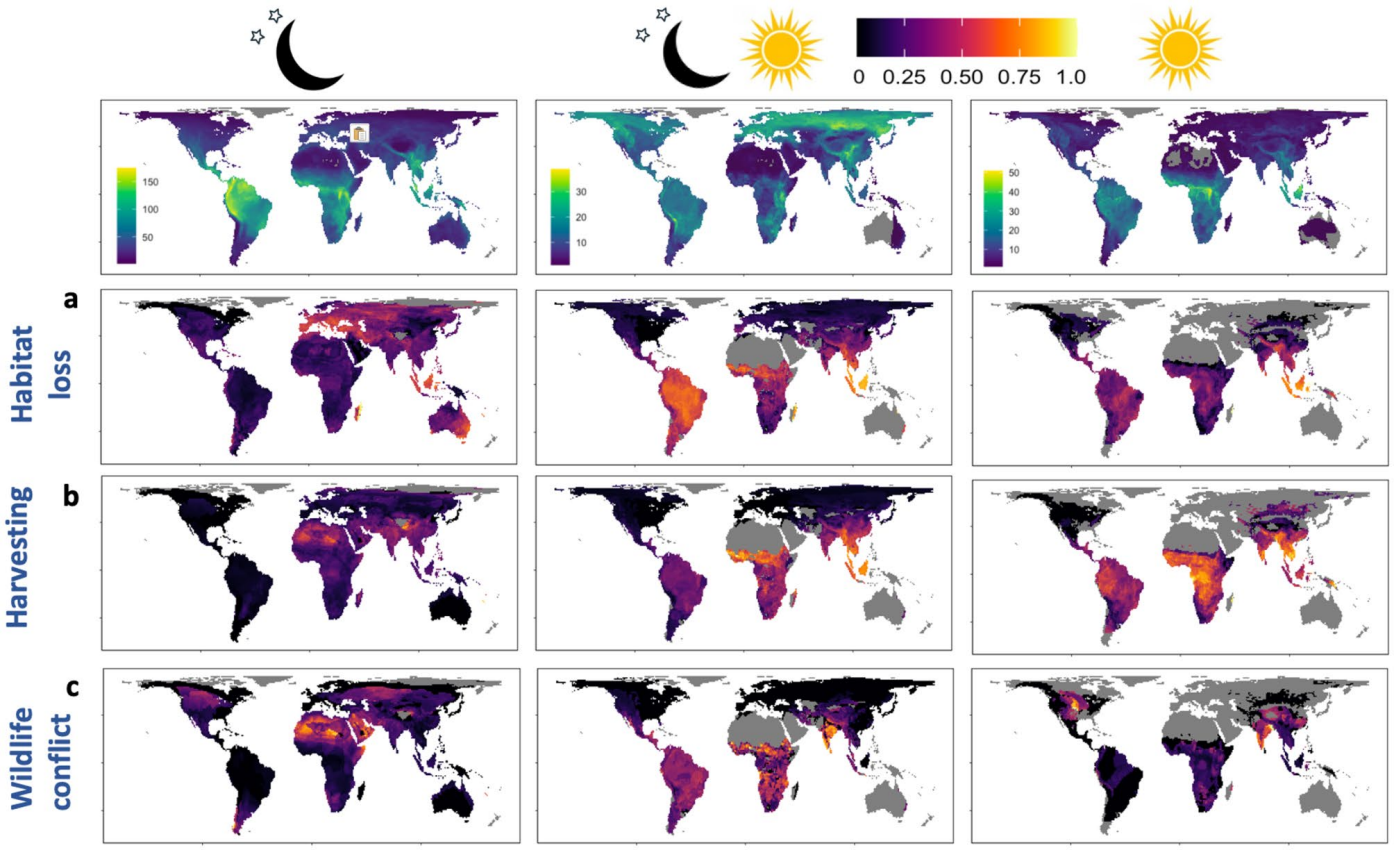
Gerographic variation in the proportion of species with declining populations in each diel niche. Based on the current IUCN range maps we show the species richness in each diel niche (top row) for nocturnal (moon and stars image), cathemeral (moon and stars and sun image) and diurnal (sun image) species. The legend gives the species richness in each pixel from 1 to the maximum. For each diel niche, subsequent rows show the proportion of species that are declining from (**a**) habitat loss, (**b**) harvesting and (**c**) human-wildlife conflict. Grey regions indicate where either five or fewer species are declining from this threat (**a**–**c**), or no species are present in that diel niche (top row). We have not included proportional declines in crepuscular species due to the low richness (≤ 5 species) across much of the land surface (but see Fig. S4).

Globally, habitat loss is the most important driver of population decline in nocturnal mammals, accounting for declines in a high proportion of species in Europe, South-East Asia, Madagascar and Australia (Fig. 3a). Where nocturnal species richness is reduced, harvesting (Sahara) and human-wildlife conflict (Sahara, Middle East, North America) are increasingly important in driving population declines (Fig. 3b,c). Across the tropics, a greater proportion of cathemeral mammals are declining than those occupying other diel niches due to a combination of habitat loss, harvesting and human-wildlife conflict. Proportionally, habitat loss is the most prevalent driver of population declines in cathemeral species (Fig. 3a), except in sub-Saharan Africa where harvesting, and India where human-wildlife conflict, are associated with declines in a higher proportion of species (Fig. 3b,c). In contrast, at the higher latitudes where cathemeral species richness is greatest, most species population trends are not currently declining (Fig. 3). Over much of the tropics a greater proportion of diurnal species are declining from harvesting than habitat loss, with the exception of Indonesia where habitat loss is associated with declines in a high proportion of species regardless of when they are active (Fig. 3a,b). In central North America human-wildlife conflict is associated with a higher proportion of declining diurnal species than other anthropogenic threats (Fig. 3c).

## Discussion

Here, we show that, associated with the larger proportion of diurnal mammals that are primates, populations of solely day-active species are more likely to be declining, and to face an increased number of threats, than those occupying other diel niches. The degradation or loss of the habitats on which species depend is the most common threat, impacting species equally, regardless of when they are active. Relative to nocturnal mammals, a greater proportion of diurnal species are declining due to harvesting than habitat loss, although many experience both threats. However, proportionally, the greatest declines are occurring in cathemeral species in the tropics associated with the vulnerability of megafauna and arboreal foragers to habitat loss, harvesting and human-wildlife conflict^21,24,25^.

Across much of the tropics, high species richness buffers nocturnal communities from species losses, so that a relatively low proportion of night-active species are declining. Most nocturnal mammals are small and cryptic and in the main avoid threats such as harvesting and human-wildlife conflict. As such, habitat loss is the sole driver of decline in the majority of species. Doubtless a consequence of variation in harvesting methods, a lower proportion of nocturnal species are declining from harvesting in the Amazon (where high-value species tend to be targeted with projectile weapons), than in sub-Saharan Africa and tropical Asia (where there is a preference for snares that indiscriminately capture a broad range of species active both day and night^26^). In Australia, where the land mammal fauna is characterised by nocturnality the drivers of population decline are dissimilar to elsewhere in the world^27^, being driven by invasive species, changing fire regimes^27^ and climate change^28^ (Fig. S4B).

Cathemerality is a common strategy in megafauna^29^ (44% of species weighing > 45 kg are cathemeral; *N* = 147), and we find that across the tropics proportionally declines from habitat loss, harvesting and human-wildlife conflict are most severe in cathemeral species. Given the high proportion of diurnal mammals that are also declining, it is likely that it is the activity of cathemeral species during the daytime that puts them at an increased risk of decline. In larger species cathemerality allows activity to be timed to when anthropogenic pressures are reduced, with many species shifting a greater proportion of their activity to the nighttime (reviewed in a meta-analysis by Gaynor et al.^30^). Nevertheless, the high proportion of cathemeral species that are threatened in the tropics suggests that individual diel flexibility is not sufficient to avoid population decline in the Anthropocene. Unlike nocturnal and diurnal species where species richness is highest in the tropics, cathemeral richness^31^ and functional diversity^10^ dominates in the upper latitudes, where in the main populations are not declining. However, the upper latitudes are warming more quickly than the rest of the planet and in the future cathemeral species located there may become increasingly vulnerable^32,33^.

Mammals (primates and non-primates) that are active mostly or solely during the daytime are increasingly likely to encounter humans (a predominantly diurnal species), making them vulnerable to both targeted and opportunistic harvesting and more likely to come into conflict with people than are night-active species. For example, although diurnal primates tend to be more flexible in their use of human modified landscapes than their nocturnal and cathemeral counterparts^34^, associated closer interactions with humans are not without risk and over half of declining populations of diurnal primates are due to harvesting. Nevertheless, overall, a slightly lower proportion of diurnal primates are in decline compared to nocturnal and cathemeral primates (75% compared to 80% and 95%, respectively). Across much of the tropics a similar proportion of all diurnal species are declining from harvesting as they are from habitat loss, suggesting that day-active mammals may be particularly vulnerable to the additive or synergistic impacts of these pressures^19,20^.

Here, we show that being active at the same time as humans increases the risk of population decline from harvesting and human-wildlife conflict and may be a risky strategy for species persistence in the Anthropocene. However, diurnality could be beneficial in the context of management and species conservation. (1) Compared to nocturnal mammals, the ecology, abundances and ranges of many day-active species are relatively well known^35^, allowing comprehensive management plans to be produced, (2) both conservation practitioners and wildlife harvesters are mostly diurnal providing increased opportunities to reduce illegal and unsustainable harvesting, (3) the opportunity to interact with day-active species can be an important focus of eco-tourism, providing greater motivation for local communities to protect wildlife. Notwithstanding, as defaunation in the Anthropocene progresses, increasingly the nighttime is likely to act as a haven for mammals.

## Methods

### Diel niche

We extracted data on the diel niche (activity pattern) of each species from our recently compiled database for mammals^9^; in light of more recent information, we updated diel niche for 108 species (Data S1). In brief, following the Handbook of Mammals of the World^36^, we assigned each species to one of four activity patterns: (1) nocturnal—active mostly or only at night; (2) crepuscular—active only during twilight (around sunrise and/or sunset); (3) cathemeral—significant activity during both the day and night; (4) diurnal—active mostly or only during the day (Data S1). We excluded sea mammals (*N* = 127; including two species of marine otter, *Enhydra lutris* and *Lontra felina*) and species described as highly or fully fossorial (*N* = 247), because these are likely to be reliant on different light cues than above surface terrestrial species. We phylogenetically imputed data on diel niche for 156 species (3%; Appendix S1).

### Population trend

To identify the direction of the population trend in each species we took several approaches. First, we downloaded known population trends for 2,926 species from the IUCN Red List and designated these as declining (i.e., decreasing), non-declining (i.e., increasing, stable) or unclassified (IUCN, 2021). In > 91% of species classed as Near-threatened (NT), Vulnerable (VU), Endangered (EN) or Critically Endangered (CR) whose population trend is known, the population is declining (i.e., populations are considered stable or increasing in < 9% of species). Therefore, we also assigned a declining population trend to NT, VU, EN or CR species with unclassified population trends (*N* = 150; Table S1). The population trend for 1288 species classed as Least Concern (LC; *N* = 2956), and 659 species classed as Data Deficient (DD; *N* = 670) were unclassified. Using published estimates of threat status and literature searches we estimated population trends in 1348 species, with the trend in 599 species (476 LC species; 123 DD species) remaining unclassified (Appendix S1). Sensitivity tests revealed that our results were robust to population trends estimated here and variation in population decline across threatened (VU, EN, CR) and non-threatened (LC, NT) species (Appendix S1).

### Anthropogenic threats

We downloaded data on major anthropogenic threats to species from Brodie et al*.*^21^ who, based on the IUCN Red List, grouped threats into ten categories (habitat loss, harvesting [e.g., hunting, poaching, snaring], human-wildlife conflict, climate change, non-native species, pollution, hybridization, prey depletion, disease, and inbreeding; Tables S4 and S5 (reproduced from Brodie et al.^32^). Anthropogenic threats were unknown for 676 species whose population trends, as defined above, are declining (20 CR species; EN 55 species; 72 VU species; 76 NT species; 191 LC species; 269 DD species). For these species, following the methodology of Brodie et al*.*^21^, and where possible, anthropogenic threats were identified from the Handbook of Mammals of the World^36^ and extensive literature searches (Data S1; *N* = 649). Anthropogenic threats remained unclassified for 1.3% of species (23 nocturnal, 1 cathemeral, 3 diurnal).

### Statistical analysis

For analysing variation in (1) population decline across diel niches, (2) the number of threats faced by declining populations across diel niches, and (3) the likelihood that populations are declining in each diel niche by threat type, we built phylogenetic generalized models^37^ using the *phyloglm()* function in the ‘phylolm’ package^38^. A phylogenetic supertree was available from the PHYLACINE 1.2.1 database for all 5032 mammal species^23^ (Appendix S1). To avoid issues of circularity^39^, we excluded species with a phylogenetically imputed diel niche classification (*N* = 156). Phylogenetic data are not available for all species, the PHYLACINE 1.2.1 database employs a hierarchical Bayesian approach to provide a posterior distribution of 1000 trees, which is intended to recover uncertainties in topology and branch length of missing species^23^. To account for uncertainty about phylogenetic topology or divergence dates in any one tree, we randomly selected 100 trees and repeated each model for each tree in turn. We present the mean coefficients across the 100 models, the standard deviation of the mean coefficients, the percentage of repetitions in which the *p* value < 0.01 and the mean pseudo R-squared.

1. We built a phylogenetic logistic regression model that optimizes the Generalized Estimating Equation (GEE) to the penalized likelihood of the logistic regression (method set to “logistic_MPLE”), of whether population trend was declining (1) or not (0), for all 4876 species, modelled against diel niche.
2. For each species with a declining population trend (*N* = 2067) we summed the number of anthropogenic threats, before building a phylogenetic GEE for Poisson regression (method set to “poisson_GEE”) of the number of threats (0–6) modelled against diel niche.
3. Finally, for species with a declining population trend (*N* = 2067), for each major anthropogenic threat (habitat loss, harvesting, human-wildlife conflict, climate change, non-native species; the remaining five threats contained less than 50 species each, and were excluded from this analysis) we built a phylogenetic logistic regression model optimized to penalize likelihood of the logistic regression (method set to “logistic_MPLE”), with a binary response of whether the species was declining due to that threat (1) or not (0), modelled against diel niche.

### Range maps

Maps of current mammalian ranges were downloaded from PHYLACINE 1.2.1^23^ as binary rasters of species presence (1) or absence (0) projected to Behrmann cylindrical equal area (*EASE-Grid 2.0: EPSG:6933*^40^ at a spatial resolution 96.5 by 96.5 km^23^. For all species, the maps include what the IUCN considers their current, natural and reintroduced ranges and excludes pixels coded as introduced, extinct or probably extinct^21^. For each diel niche and each of the five main anthropogenic threats (habitat loss, harvesting, human-wildlife conflict, climate change, non-native species) we then summed the number of species with declining populations, and present these as a proportion of the total number of species in that diel niche. To avoid pixels with a low species richness biasing the maps, pixels with ≤ 5 species in a given diel niche were excluded (coloured grey). Following Brodie et al.^21^ the default assumption was that a declining species was declining throughout its range (Appendix S1). However, for the spatial analysis we then went through the text descriptions in each IUCN species account and added species back to countries where they were known not to be in decline. For example, the Western Barbastelle *Barbastella barbastellus* is declining at species level, but not in certain range countries (e.g., Germany, Ukraine).

### Sensitivity tests

Overall, our results and conclusions were qualitatively similar (1) with regards to testing primate and non-primate mammals separately, compared to all species combined. For the proportion of declining species compare Fig. 1A with Fig. S1 (Table S1); for the number of threats faced by species with declining populations compare Figs. 1B and S2 (Table S2); for the threats faced by declining populations compare Fig. 2A with Fig. S3 (Table S2). (2) including and excluding estimated population trends (compare Tables S2 and S5 with Table S6). (3) with variation in population decline between threatened (VU, EN, CR) and non-threatened (LC, NT) species (non-threatened species were considered to have a non-declining population trend; compare Tables S2 and S3 with Table S7).

## Supplementary Information

Supplementary Information 1.

Supplementary Information 2.

## Data Availability

The datasets generated and analysed during the current study are available in the supplementary information (Data S1). Phylogenetic data and range maps were downloaded from PHYLACINE 1.2.1 and are available on the Dryad Digital Data Repository (https://doi.org/10.5061/dryad.bp26v20).
