# Supplementary material for: Diel niche variation in mammalian declines in the Anthropocene
Source: Sci Rep. 2023 Jan 19;13:1031. doi: 10.1038/s41598-023-28104-2 (PMC9852540; doi:10.1038/s41598-023-28104-2)
Supplement: Supplementary file 2 — Supplementary Information 2. [file 41598_2023_28104_MOESM2_ESM.docx]

**Diel niche variation in mammalian declines in the Anthropocene**

Daniel T.C. Cox *, Alexandra S. Gardner, Kevin J. Gaston

Environment and Sustainability Institute, University of Exeter, Penryn, Cornwall, TR10 9FE, U.K.

*Corresponding author: Daniel TC Cox: [d.t.c.cox@exeter.ac.uk](mailto:d.t.c.cox@exeter.ac.uk); Phone: +44 (0) 7800556070

**Appendix S1:** Supplementary methodology.

**Table S1:** Diel variation in population decline from anthropogenic threats.

**Table S2:** Phylogenetic Generalised Linear Models for the proportion of declining species and the number of threats faced by species with declining populations.

**Table S3:** Phylogenetic logistic regression of variation in the proportion of declining species in each diel niche associated with five anthropogenic threats.

**Table S4:** Population trends pooled across diel niches.

**Table S5:** Anthropogenic threat categories.

**Table S6:** Sensitivity test 1: estimated population trends.

**Table S7:** Sensitivity test 2: threatened vs non-threated species.

**Fig. S1:** The proportion of (A) primates, and (B) non-primates with declining population trends by diel niche.

**Fig. S2:** The number of threats faced by species with declining populations for (A) primates, and (B) non-primates, by diel niche.

**Fig. S3:** The proportion of declining (A) primate, and (B) non-primate mammals from five anthropogenic threats.

**Fig. S4:** Biogeographic variation in the proportion of threatened species from anthropogenic threats.

**Appendix S1:** Supplementary methodology

*Diel niche*

Data on diel niche were not available for 156 species (3%). To achieve complete species-trait coverage we imputed missing data using the Multivariate Imputation with Chained Equations (MICE) package based on the ecological (diel niche) and phylogenetic (the first ten phylogenetic eigenvectors extracted from trees obtained from PHYLACINE 1.2 database^1-2^ relationships between species. We then selected at random one of the 25 possible datasets extracted from data imputation^3^ and used this for all analyses (dataset 24; Data S1). The reliability of the imputed data compared to a data-deletion approach has been previously validated (Cox et al., 2021). All data processing and analyses were performed in R software for statistical computing v4.1.2 (R Core Team, 2019).

*Population trend*

We estimated unclassified population trends in 1,288 species classed as Least Concern (LC; *N* = 2,956), and 659 species classed as Data deficient (DD; *N* = 670). To estimate the population trends in these species we searched the Handbook of Mammals of the World^4^, the most comprehensive resource available for mammalian macroecological data covering all mammal species at the time of writing (published 2001-2019). Text information is provided for each species on its ‘conservation and status’. The population trend was designated as non-declining if populations were widely distributed and presumed to have a large population, or had no known major threats, or had populations within protected areas that were considered non-declining (*N* = 757). Species with major threats and whose populations are declining were considered to be declining (*N* = 54). The population trend of species whose only indication of its conservation status was its IUCN threat status as LC, remained unclassified *(N =* 476).

Six-hundred and seventy species were classed by IUCN as Data Deficient (DD), with the population trend being unclassified for 659 of these. For 486 DD species we used published estimated classifications of threat status^5-6^. These studies classed species as threatened or non-threatened based on functional traits, rarity (geographical range size) and range loss (human encroachment on species range), together with phylogenetic and spatial dependencies^5-6^. Although it is not known whether populations of threatened species are currently declining, as above where population trends are known they are declining in >92% of threatened species and therefore here we considered populations of threatened species to be declining (*N* = 313; Table S4). Conversely, populations of non-threatened species were classed as non-declining (*N* = 173). Following the methodology above we identified population trends of a further 49 species in the Handbook of Mammals of the World^4^ (Data S1). Overall, the population trend in 599 species (476 LC species; 123 DD species) remained unclassified. As exclusion of these species reduces sample sizes (and consequently the statistical power of the analysis) and may introduce bias^2,7^, we took a conservative approach and considered their population trends to be non-declining.

*Sensitivity tests*

Overall, our results and conclusions were qualitatively similar (1) with regards to testing primate and non-primate mammals separately, compared to all species combined. For the proportion of declining species compare Fig. 1A with Fig. S1 (Table S1); for the number of threats faced by species with declining populations compare Fig. 1B and Fig. S2 (Table S2); for the threats faced by declining populations compare Fig. 2A with Fig. S3 (Table S2). (2) including and excluding estimated population trends (compare Table S2 and S5 with Table S6). (3) variation in population decline between threatened (VU, EN, CR) and non-threatened (LC, NT) species (non-threatened species were considered to have a non-declining population trend; compare Table S2 and S3 with Table S7).

*Statistical analysis*

Related species tend to resemble one another, therefore it is preferable to control for phylogenetic signal^8^. Inclusion of phylogenetically imputed traits in phylogenetic models can cause issues due to circularity^2^, therefore we excluded species with phylogenetically imputed diel niche from the threat analyses (n = 156). Phylogenetic data are not available for all species, the PHYLACINE 1.2.1 database employs a hierarchical Bayesian approach to provide a posterior distribution of 1000 trees, which is intended to recover uncertainties in topology and branch length of missing species^1^. To account for uncertainty about phylogenetic topology or divergence dates in any one tree, we randomly selected 100 trees and repeated each model, for each tree in turn. We present the mean coefficients across the 100 models, the standard deviation of the mean coefficients, the percentage of repetitions in which the *p* value <0.01 and the mean pseudo R-squared.

*Range maps*

Maps of current mammalian ranges were downloaded from PHYLACINE 1.2.1^1^. The maps were created from IUCN Version 2016-3 and were available as binary rasters of species presence (1) or absence (0) projected to Behrmann cylindrical equal area (*EASE-Grid 2.0: EPSG:6933^9^*) at a resolution 96.5 by 96.5 km^1^. For all species, the maps include what the IUCN considers their current, natural and reintroduced ranges and excludes pixels coded as introduced, extinct or probably extinct^1^.

**References**

1. Faurby, S., Davis, M., Pedersen, R. Ø., Schowanek, S. D., Antonelli, A., & Svenning, J.C.. PHYLACINE 1.2.1: The phylogenetic atlas of mammal macroecology. *Ecology* **99**, 2626–2626 (2018). https://doi.org/10.1002/ecy.2443.
2. Penone, C., Davidson, A. D., Shoemaker, T. E., Di Marco, M., Rondinini, C., Brooks, T. M…. Costa, G. C.. Imputation of missing data in life-history trait datasets: which approach performs the best? *Methods in Ecology and Evolution* **5**, 961–70 (2014). https://doi.org/10.1111/2041-210X.12232.
3. Cox, D. T. C., Gardner, A. S. & Gaston, K. J. Diel niche variation in mammals associated with expanded trait space’. *Nature Communications* **12**, 1753 (2021). https://doi.org/10.1038/s41467-021-22023-4.
4. Mittermeier, R., Rylands, A. B., Lacher, T. E., & Wilson, T. E. *Handbook of the Mammals of the World - Volumes 1-3 & 5-9*. Lynx Edicions (2001-2019).
5. Bland, L. M., Collen, B. C., Orme, D. L. & Bielby, J. Predicting the conservation status of data-deficient species. *Conservation Biology* **29**, 250–59 (2015). https://doi.org/10.1111/cobi.12372.
6. Jetz, W. & Freckleton, R. P. Towards a general framework for predicting threat status of data-deficient species from phylogenetic, spatial and environmental information. *Philosophical Transactions of the Royal Society B: Biological Sciences* **370**, 20140016 (2015). https://doi.org/10.1098/rstb.2014.0016.
7. Taugourdeau, S., Villerd, J., Plantureux, S., Huguenin-Elie, O., & Amiaud, B. Filling the gap in functional trait databases: use of ecological hypotheses to replace missing data. *Ecology and Evolution* **4**, 944–58 (2014). https://doi.org/10.1002/ece3.989.

**Table S1:** Diel variation in population decline from anthropogenic threats. We show (A) the number of species experiencing population declines in each diel niche, (B) the number of anthropogenic threats faced by species with declining populations in each diel niche, and (C) the number of species experiencing declines from the five most prevalent anthropogenic threats. For (B) we also give the number of declining species in the four orders for which richness is comparable across diel niches. Parentheses for (A) are the percentage of all species, and parentheses for (B-C) are the percentage of declining species. Data underpinning (A) Fig. 1A and Fig. S1, (B) Fig. 1B and Fig. S2, and (C) Fig. 2A.

|  | Nocturnal | Crepuscular | Cathemeral | Diurnal |
| --- | --- | --- | --- | --- |
| (A) Declining | populations |  |  |  |
| *N* | 3,498 | 113 | 526 | 895 |
| All species | 1,402 (40.1%) | 45 (39.8%) | 226 (43.0%) | 466 (52.1%) |
| Primates | 93 (80.2%) | 0 | 20 (95.2%) | 217 (75.6%) |
| Non-primates | 1,309 (38.7%) | 45 (39.8%) | 206 (40.8%) | 249 (41.0%) |
|  |  |  |  |  |
| (B) |  |  |  |  |
| Threats unknown | 22 (1.6%) | 0 (0%) | 1 (0.4%) | 3 (0.6%) |
| 1 | 1075 (76.7%) | 23 (51.1%) | 131 (58.0%) | 240 (51.5%) |
| 2 | 256 (18.3%) | 18 (40.0%) | 71 (1.4%) | 178 (38.2%) |
| 3 | 39 (2.8%) | 4 (11.8%) | 13 (5.8%) | 35 (7.5%) |
| ≥4 | 10 (0.7%) | 0 (0%) | 10 (4.4%) | 10 (2.1%) |
| *Facing ≥2 (≥3) threats* |  |  |  |  |
| Primates | 22 (0) | 0 (0) | 9 (0) | 105 (16) |
| Carnivores | 38 (11) | 1 (0) | 30 (11) | 17 (10) |
| Artiodactyla | 18 (3) | 13 (3) | 30 (6) | 48 (9) |
| Rodentia | 74 (11) | 1 (0) | 4 (1) | 23 (8) |
|  |  |  |  |  |
| (C) *Threats* |  |  |  |  |
| Habitat loss | 1,279 (91.2%) | 42 (87.5%) | 202 (89.0%) | 395 (85.5%) |
| Harvesting | 215 (15.3%) | 23 (47.9%) | 87 (38.3%) | 239 (51.7%) |
| Wildlife conflict | 37 (2.6%) | 2 (4.2%) | 18 (7.9%) | 30 (6.5%) |
| Climate change | 26 (1.9%) | 2 (4.2%) | 10 (4.4%) | 9 (2.0%) |
| Non-natives | 120 (3.4%) | 4 (3.4%) | 13 (2.5%) | 27 (3.0%) |
| Other^#^ | 70 (5.0%) | 2 (4.2%) | 36 (15.9%) | 30 (6.5%) |

^#^ Other major threats are pollution, hybridization, prey depletion, disease, inbreeding

**Table S2:** Phylogenetic Generalised Linear Models of variation across diel niches in (A) the proportion of species that are experiencing population declines, and (B) the number of anthropogenic threats faced by species with declining populations. To test whether results were biased by the high proportion of diurnal primates, we repeated (A) and (B) for primates only and non-primates only. To account for uncertainty about phylogenetic topology or divergence dates in any one phylogenetic tree, we randomly selected 100 trees and repeated the model for each anthropogenic treat for each tree in turn. We present the mean coefficients across the 100 models for each analysis, the standard deviation of the coefficients across models (in parentheses), the percentage of repetitions in which the *p* value <0.01, where; ### is ≥90% of models, ## is ≥75% and <90% of models, # ≥60% and <75% of models), and the mean *pR^2^*.

|  | All species | Primates | Non-primates |
| --- | --- | --- | --- |
| 1. *Proportion declining* | |  |  |
| Intercept | -0.6 (±0.3)^###^ | 1.2 (±0.5) | -0.5 (±0.3)^###^ |
| Crepuscular | 0.3 (±0.3) | NA | 0.04 (±0.3) |
| Cathemeral | 0.6 (±0.4) ^#^ | 0.9 (±0.6) | 0.1 (±0.1) |
| Diurnal | 0.8 (±0.4) ^###^ | -0.5 (±0.3) | 0.2 (±0.2) |
| pR2 | <0.01 | 0.28 | <0.01 |
|  |  |  |  |
| 1. *Number of threats* | |  |  |
| Intercept | 0.5 (±0.04) | 0.3 (±0.1) | 0.4 (±0.03) |
| Crepuscular | -0.1 (±0.2)^##^ | NA | -0.01 (±0.1)## |
| Cathemeral | 0.04 (±0.1)^##^ | 0.02 (±0.02) | 0.01 (±0.08)## |
| Diurnal | 0.1 (±0.1)^###^ | 0.3 (±0.02)### | 0.1 (0.1)### |

**Table S3:** (A) Phylogenetic logistic regression of variation in the proportion of declining species in each diel niche associated with five anthropogenic threats (*N* = 2,067). To account for uncertainty about phylogenetic topology or divergence dates in any one phylogenetic tree, we randomly selected 100 trees and repeated the model for each anthropogenic treat for each tree in turn. We present the mean coefficients across the 100 models for each analysis and the standard deviation of the mean coefficients (in parentheses). The percentage of repetitions where a *p* value <0.01 is shown, where # ≥60%-<75%, ## ≥75%-<90% and ### ≥90% of models. We also give the mean *pseudo R-*squared across models. We also show (B) Anthropogenic threats across diel niches in primates with declining populations (*N* = 322), and (C) Anthropogenic threats across diel niches in non-primates with declining populations (*N* = 1,745).

|  | Habitat loss | Harvesting | Wildlife Conflict | Climate change | Non-natives |
| --- | --- | --- | --- | --- | --- |
| (A) |  |  |  |  |  |
| Intercept | 2.4 (_­_±0.04)### | -1.9 (±0.01)### | -3.4 (±0.01)### | -4.1 (±0.02)### | -2.3 (±0.01)### |
| Crepuscular | -0.5 (±0.03) | 0.3 (±0.03) | 0.3 (±0.1) | 1.3 (±0.04) | 0.2 (±0.01) |
| Cathemeral | -0.5 (±0.01) | 0.4 (±0.01) | 0.4 (±0.02) | 1.2 (±0.01)### | -0.6 (±0.02) |
| Diurnal | -0.7 (0.005)### | 1.39 (±0.01)### | 1.0 (±0.01)### | 0.4 (±0.02) | -0.6 (±0.01) |
| *pR^2^* | 0.02 | 0.46 | 0.09 | 0.06 | 0.12 |
|  |  |  |  |  |  |
| *(B) Primates only* | |  |  |  |  |
| Intercept | 1.4 (±0.4) | -1.2 (±0.05)### | -10.2 (±4.2) | -9.0 (±4.1) | -2.5 (±1.0) |
| Cathemeral | 3.5 (±6.5) | 0.7 (±0.2) | 5.8 (±3.1) | 5.2 (±3,4) | 0.3 (±0.3) |
| Diurnal | -1.3 (±0.3)# | 1.8 (±0.1)### | 8.0 (±3.9) | 5.1 (±4.0) | 0.5 (±0.3) |
| *pR^2^* | 0.08 | 0.21 | 0.13 | <0.01 | 0.03 |
|  |  |  |  |  |  |
| *(C) Non-primates only* | |  |  |  |  |
| Intercept | 2.2 (±0.04)### | -2.0 (±0.09)### | -3.3 (±0.1)### | -4.0 (±0.1)### | -2.1 (±0.1)### |
| Crepuscular | -0.6 (±0.2) | 0.2 (±0.2) | 0.5 (±0.2) | 1.4 (±0.2) | 0.1 (±0.2) |
| Cathemeral | -0.6 (±0.1) | 0.4 (±0.07) | 0.3 (±0.2) | 0.01 (±0.01) | -0.7 (±0.3) |
| Diurnal | -0.6 (±0.1)# | 1.1 (±0.08)### | 0.7 (0.2) | 0.2 (0.1) | -0.4 (0.1) |
| *pR^2^* | 0.02 | 0.49 | 0.9 | 0.06 | 0.10 |

**Table S4:** Population trends pooled across diel niches as identified by Brodie et al. (2021). We show the percentage of species where the population trend is unknown, and from those that are known population trends that were classed as decreasing. Population trends are indicated as: Least Concern (LC); Near-threatened (NT), Vulnerable (VU), Endangered (EN); Critically Endangered (CR) and Data Deficient (DD).

| IUCN status | Decreasing | Increasing | Stable | Unknown | % Decreasing | % Unknown |
| --- | --- | --- | --- | --- | --- | --- |
| *All species* |  |  |  |  |  |  |
| LC | 398 | 49 | 1200 | 1309 | 24.2 | 44.3 |
| NT | 254 | 5 | 18 | 51 | 91.7 | 15.5 |
| VU | 402 | 6 | 29 | 53 | 92.0 | 10.8 |
| EN | 374 | 6 | 6 | 37 | 96.9 | 8.7 |
| CR | 146 | 3 | 8 | 9 | 95.0 | 5.4 |
| DD | 46 | 0 | 6 | 627 | 88.5 | 92.3 |
|  |  |  |  |  |  |  |
| *Nocturnal* |  |  |  |  |  |  |
| LC | 245 | 18 | 850 | 998 | 22.0 | 47.3 |
| NT | 152 | 3 | 13 | 42 | 90.5 | 20.0 |
| VU | 225 | 1 | 21 | 47 | 91.1 | 16.0 |
| EN | 224 | 2 | 3 | 32 | 97.8 | 12.3 |
| CR | 72 | 2 | 2 | 11 | 94.7 | 12.6 |
| DD | 31 | 0 | 3 | 506 | 91.2 | 93.7 |
|  |  |  |  |  |  |  |
| *Crepuscular* |  |  |  |  |  |  |
| LC | 8 | 3 | 25 | 37 | 22.2 | 50.7 |
| NT | 8 | 1 | 1 | 3 | 80.0 | 23.1 |
| VU | 13 | 0 | 0 | 0 | 100 | 0 |
| EN | 12 | 1 | 0 | 0 | 92.3 | 0 |
| CR | 3 | 0 | 0 | 0 | 100 | 0 |
| DD | 2 | 0 | 1 | 6 | 66.7 | 66.7 |
|  |  |  |  |  |  |  |
| *Cathemeral* |  |  |  |  |  |  |
| LC | 44 | 14 | 131 | 96 | 23.3 | 33.7 |
| NT | 20 | 0 | 1 | 5 | 95.2 | 19.2 |
| VU | 33 | 3 | 4 | 4 | 82.5 | 9.1 |
| EN | 29 | 1 | 1 | 5 | 93.5 | 13.9 |
| CR | 16 | 1 | 0 | 1 | 94.2 | 5.6 |
| DD | 16 | 1 | 0 | 1 | 94.2 | 5.6 |
|  |  |  |  |  |  |  |
| *Diurnal* |  |  |  |  |  |  |
| LC | 101 | 14 | 194 | 178 | 32.7 | 36.6 |
| NT | 68 | 1 | 1 | 9 | 97.1 | 11.4 |
| VU | 94 | 1 | 4 | 11 | 94.9 | 0.1 |
| EN | 104 | 2 | 1 | 6 | 97.2 | 5.3 |
| CR | 52 | 0 | 3 | 3 | 94.5 | 5.2 |
| DD | 6 | 0 | 1 | 61 | 85.7 | 89.7 |

**Table S5:** Anthropogenic threat categories. Reproduced from Brodie et al., (2021),

| Threat | Explanation |
| --- | --- |
| Habitat loss | This includes actual loss as well as habitat fragmentation and habitat degradation. Mechanisms are variable, but include deforestation, agricultural expansion, livestock grazing, mining, human-caused fires, roads (and collisions with vehicles), loss of habitat connectivity, etc. In many cases the word ‘habitat’ was not used in the IUCN species account explicitly, but was inferred from context (e.g., “draining of wetlands”, “expansion of agriculture”, “selective logging”, “clear-cutting”). |
| Harvesting | This category includes legal and illegal exploitation in the form of direct hunting, snaring, and netting (all for a variety of subsistence and/or market-based reasons), as well as indirect bycatch in snares or nets that were set out to catch other species. |
| Human-wildlife conflict | This is where humans kill animals to protect crops, livestock, or themselves or to remove animals that are above some (usually implicit) societal threshold in tolerable abundance. Terms in the IUCN species accounts included “retaliatory killing”, “crop raiding”, “agricultural pest”, and “cull”. This also includes carcass poisoning (to kill scavenging carnivores) and pesticides to kill rodents. Note that non-target effects of pesticides (e.g. rodenticides that inadvertently kill carnivores) were classified as Pollution rather than Human-wildlife conflict. |
| Climate change | This was associated with phrases such as “warming” or “loss of sea ice” in the IUCN species accounts. Climate change was often listed as a speculative threat (e.g. “...climate change could be an additional threat...”), in which case it was not judged to be a major cause of population decline. |
| Non-native species | This includes competition from or predation by invasive species, including free-ranging feral animals such as dogs. Note that competition from non-native livestock on pasture lands was classified as Habitat Loss. |
| Pollution | This includes chemical pollutants, poisoning by fertilizers, and mechanical pollutions such as entanglement with debris. Note that being caught in nets or snares that were targeting other species was counted as Harvest. |
| Hybridization | Some species are threatened by hybridizing with introduced or native species. |
| Prey depletion | Certain carnivores are threatened by loss of their prey. This category would also apply to specialized herbivores that were threatened by loss of their particular food plant(s). |
| Disease | Many species are subject to diseases, but in order for this to be considered a major threat to the species, the pathogen(s) had to be likely affecting the mammal’s population trend. |
| Inbreeding | Some species are demographically threatened (i.e., leading to population decline) by inbreeding depression. |

**Table S6:** Sensitivity test 1: estimated population trends. We excluded species with estimated population trends and repeated the phylogenetic generalised linear models for (A) the proportion of declining species in each diel niche (*N =* 2,927), (B) the number of threats faced by species with declining populations (*N =* 1,537), and (C) the proportion of declining species by major anthropogenic threat (*N =* 1,537). To account for uncertainty about phylogenetic topology or divergence dates in any one phylogenetic tree, we randomly selected 100 trees and repeated each model for each tree in turn. We present the mean coefficients across the models for each analysis, the standard deviation of the coefficients across models (in parentheses), the percentage of repetitions in which the *p* value <0.01 (where ### is ≥90%; ## is ≥75% to <90%; and # is ≥60% to <75% of models), and the mean pseudo R-squared.

|  | **Intercept** | **Crepuscular** | **Cathemeral** | **Diurnal** |
| --- | --- | --- | --- | --- |
| *(A) Population trend* | 0.01 (±0.01) | 0.7 (±0.1)## | 0.6 (±0.1)### | 0.9 (0.1)### |
| *pR^2^* |  |  |  |  |
|  |  |  |  |  |
| *(B) No. threats* | 0.09 (±0.4) | -0.1 (±0.1)## | 0.08 (±0.07)### | 0.13 (±0.1)### |
|  |  |  |  |  |
| *(C) Threat type* |  |  |  |  |
| *Habitat loss* | 2.2 (±0.03)### | -0.5 (±0.07) | -0.2 (±0.070 | -0.4 (±0.06) |
| *pR^2^* | <0.01 |  |  |  |
| *Harvesting* | -1.7 (±0.1)### | 0.5 (±0.2) | 0.6 (±0.2)# | 1.2 (±0.1)### |
| *pR^2^* | 0.42 |  |  |  |
| *Wildlife conflict* | -3.3 (±0.1)### | 0.7 (±0.2) | 0.4 (±0.1) | 1.1 (±0.1)### |
| *pR^2^* | 0.08 |  |  |  |
| *Climate change* | -4.0 (±0.1)### | -8.4 (±6.4) | 1.2 (±0.1)# | 0.3 (±0.2) |
| *pR^2^* | 0.08 |  |  |  |
| *Non-native sp.* | -2.1 (±0.05)### | -1.3 (±0.1) | -0.8 (±0.1) | -0.5 (0.07) |
| *pR^2^* | 0.15 |  |  |  |

**Table S7:** Sensitivity test 2: threatened vs non-threated species. We considered all non-threatened species (LC, NT) to have non-declining population trends, before repeating the phylogenetic generalised linear models for (A) the proportion of declining species in each diel niche (*N =* 4,876), (B) the number of threats faced by species with declining populations (*N =* 1,288), and (C) the proportion of declining species by major anthropogenic threat (*N =* 1,288). To account for uncertainty about phylogenetic topology or divergence dates in any one phylogenetic tree, we randomly selected 100 trees and repeated each model for each tree in turn. We present the mean coefficients across the 100 models for each analysis, the standard deviation of the mean coefficients across models (in parentheses), the percentage of repetitions in which the *p* value <0.01 (where ### is ≥90%; ## is ≥75% to <90%; and # is ≥60% to <75% of models), and the mean pseudo R-squared.

|  | **Intercept** | **Crepuscular** | **Cathemeral** | **Diurnal** |
| --- | --- | --- | --- | --- |
| *(A) Population trend* | -1.2 (±0.1)### | 0.3 (±0.1) | 0.3 (±0.3) | 0.7 (0.2)### |
| *pR2* | <0.01 |  |  |  |
|  |  |  |  |  |
| *(B) No. threats* | 0.4 (±0.2) | -0.2 (±0.2)### | 0.05 (±0.07)## | 0.08 (±0.1)## |
|  |  |  |  |  |
| *(C) Threat type* |  |  |  |  |
| *Habitat loss* | 2.6 (±0.08)### | -1.1 (±0.2) | -0.6 (±0.2) | -0.4 (±0.2) |
| *pR2* | 0.02 |  |  |  |
| *Harvesting* | -2.3 (±0.1)### | 1.3 (±0.3)# | 0.8 (±0.2)# | 1.4 (±0.1)### |
| *pR2* | 0.48 |  |  |  |
| *Wildlife conflict* | -3.6 (±0.1)### | 1.1 (±0.1) | 0.5 (±0.1) | 1.2 (±0.1)### |
| *pR2* | 0.13 |  |  |  |
| *Climate change* | -3.9 (±0.4)### | -10.2 (±5.0) | 0.7 (±0.3) | 0.3 (±0.2) |
| *pR2* | <0.01 |  |  |  |
| *Non-native sp.* | -1.9 (±0.3)### | -1.3 (±0.2) | -0.9 (±0.2) | -0.7 (0.1) |
| *pR2* | 0.09 |  |  |  |

***
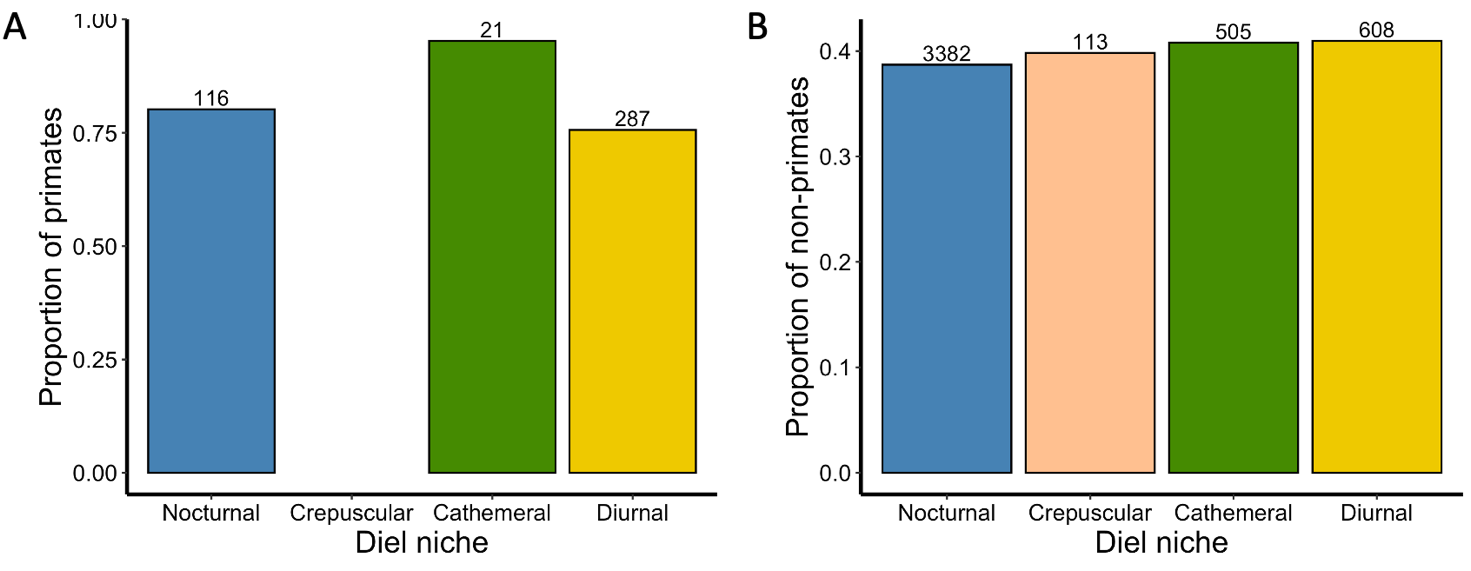
***

**Fig S1:** The proportion of (A) primates, and (B) non-primates with declining population trends by diel niche. The number above each bar gives the number of species in each group. See Table S1 and S2.


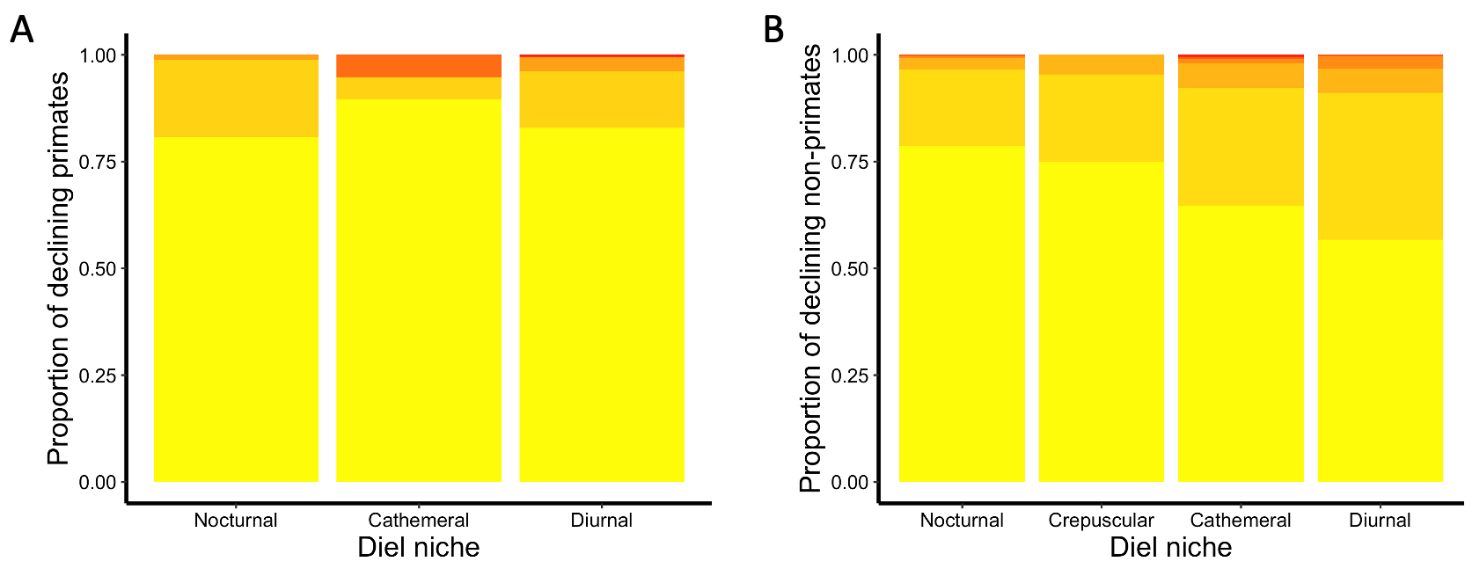


**Fig. S2:** The number of threats faced by species with declining populations for (A) primates, and (B) non-primates, by diel niche. See Table S2 for results of Phylogenetic Estimating Equations.


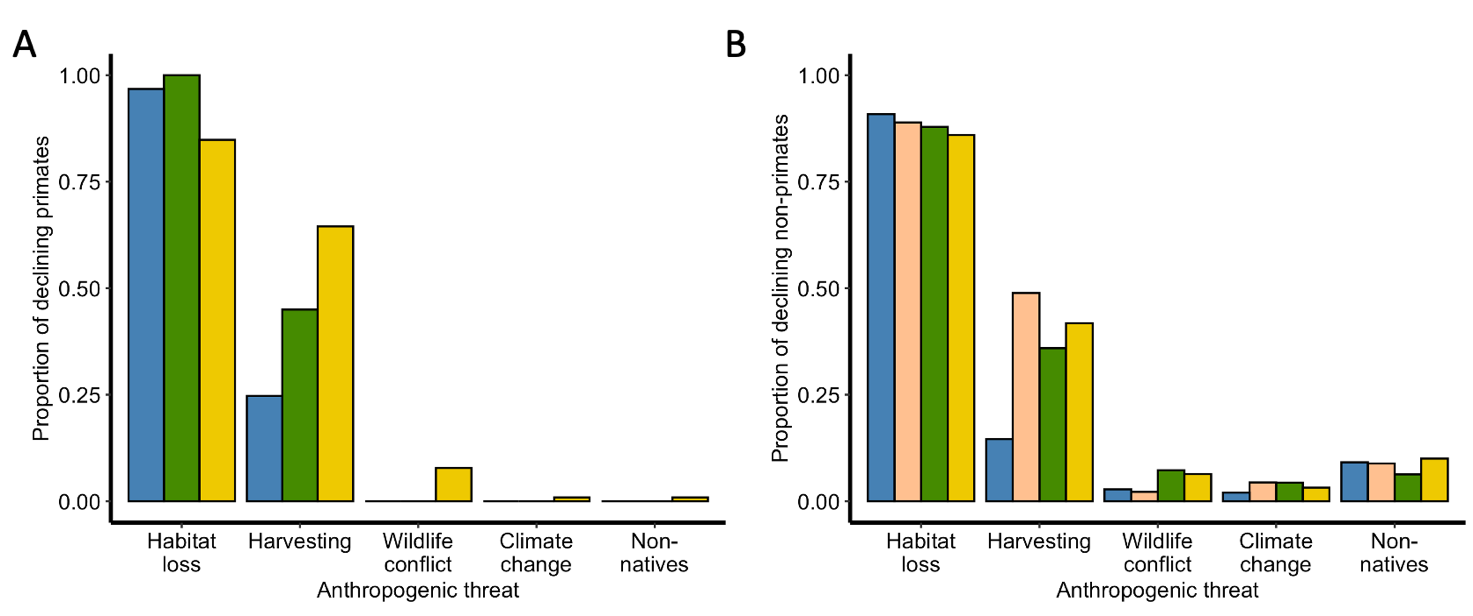


**Fig. S3:** The proportion of declining (A) primate, and (B) non-primate mammals from habitat loss, harvesting, wildlife conflict, climate change and non-native species for nocturnal (blue), crepuscular (peach), cathemeral (green) and diurnal (yellow) species. See Table S3 for results of Phylogenetic Estimating Equations.

**
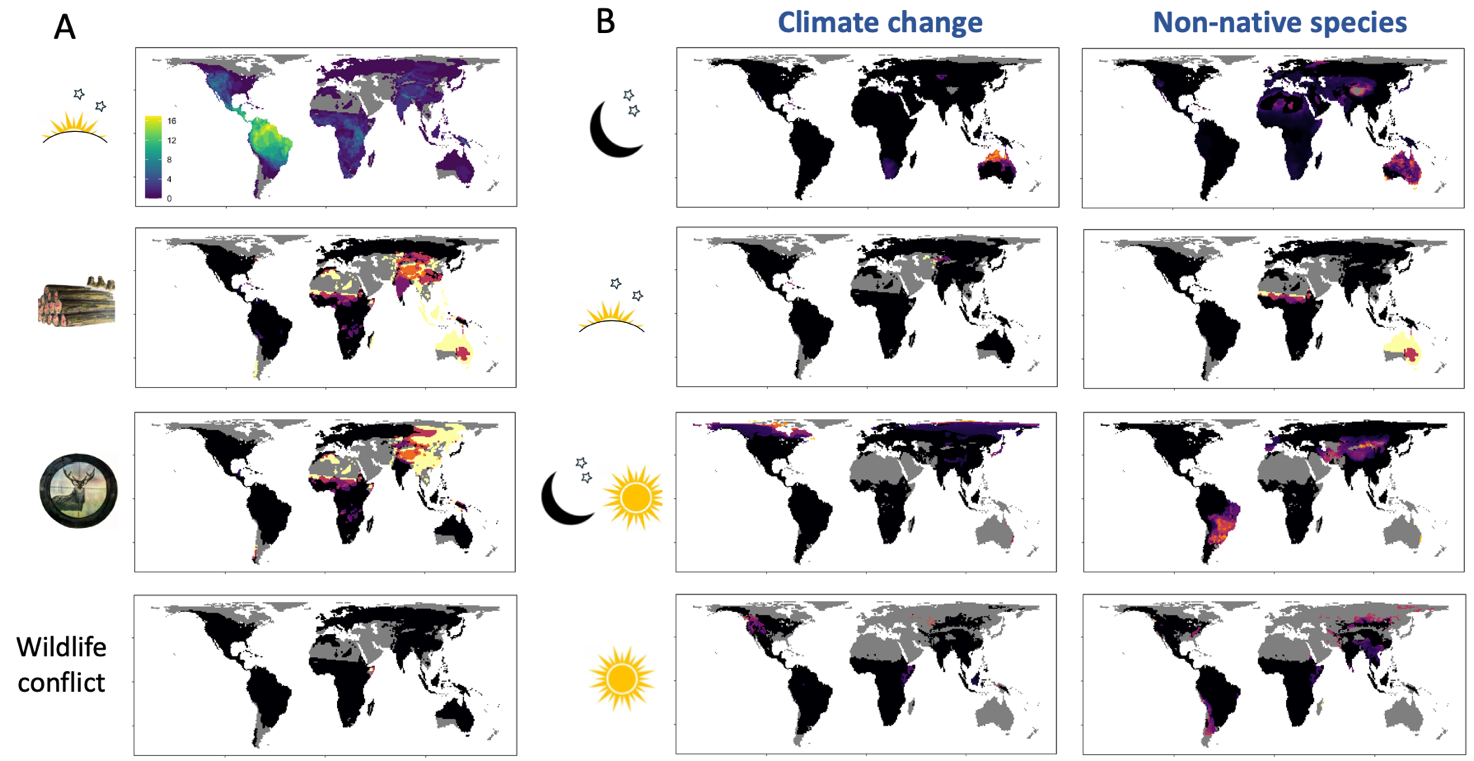
Fig. S4:** Biogeographic variation in the proportion of species with declining populations associated with five anthropogenic threats. (A) The top panel shows the species richness of current ranges for crepuscular mammals, the subsequent panels give the proportion of crepuscular species that are declining from habitat loss, harvesting and human-wildlife conflict. (B) The proportion of nocturnal (moon and stars silhouette), crepuscular (sunrise image), cathemeral (moon and stars and sun image) and diurnal (sun image) species that are declining due to climate change and non-native species. Grey shows where there are no species in that diel niche (except for nocturnal, cathemeral and diurnal maps where grey also includes <6 species as Fig. 3).
